# Hyperpolarization by Optogenetic Activation of NpHR Channels Promotes Osteogenic Differentiation of Human Dental Follicle Stem Cells

**DOI:** 10.3390/membranes16070230

**Published:** 2026-07-02

**Authors:** Dan Yang, Yuyang Luo, Fengxia Huang, Lin Hu, Xinyi Deng, Shuqi Zhang, Dongchuan Zuo, Jin Zeng

**Affiliations:** 1 Department of Orthodontics, The Affiliated Stomatology Hospital of Southwest Medical University, Luzhou 646000, China; 18764166571@163.com (D.Y.); 16677772849@163.com (Y.L.);; 2 Luzhou Key Laboratory of Oral & Maxillofacial Reconstruction and Regeneration, The Affiliated Stomatology Hospital of Southwest Medical University, Luzhou 646000, China; 15883063330@163.com (X.D.); 18989130681@163.com (S.Z.); 3 The Key Laboratory of Medical Electrophysiology, Ministry of Education, Collaborative Innovation Center for Prevention and Treatment of Cardiovascular Disease, Institute of Cardiovascular Research, Southwest Medical University, Luzhou 646000, China; 20254099120020@stu.swmu.edu.cn

**Keywords:** human dental follicle stem cells, osteogenic differentiation, membrane hyperpolarization, NpHR channel

## Abstract

Background: Membrane potential represents one of the fundamental physiological characteristics of cells, playing a critical role in cellular function. Studies have shown that membrane hyperpolarization positively regulates the osteogenic differentiation of mesenchymal stem cells. Optogenetic technology based on the Natronomonas pharaonis halorhodopsin (NpHR) light-activated channel can induce membrane hyperpolarization through optical methods. Given the working principle of optogenetic technology, this study aimed to investigate whether optogenetic activation of NpHR channels could induce membrane hyperpolarization in human dental follicle stem cells (hDFCs)—mesenchymal stem cells derived from dental follicle tissue—to regulate their osteogenic differentiation. Methods: hDFCs were isolated and cultured. Engineered hDFCs expressing the NpHR channels were constructed through lentiviral transduction. Patch clamps were performed to observe the effects of optogenetic activation of NpHR channels on membrane potentials of hDFCs. Single-cell Ca^2+^ imaging were performed to observe the effects of membrane hyperpolarization via modulation of extracellular K^+^ concentration ([K^+^]_e_) on the intracellular Ca^2+^ levels of hDFCs. Cell viability assay, transwell chamber assay, wound healing assay, osteogenic differentiation induction, alizarin red staining, alkaline phosphatase (ALP) staining, real-time reverse transcriptase polymerase chain reaction (RT-qPCR) and Western blot (WB) were performed to observe the effects of optogenetic activation of NpHR channels on proliferation, migration, and osteogenic differentiation of NpHR-hDFCs. Results: Reversing membrane hyperpolarization via modulation of extracellular K^+^ concentration ([K^+^]_e_) was shown to suppress osteogenic differentiation of hDFCs, whereas promoting membrane hyperpolarization via opening ATP-sensitive K^+^ channels was shown to enhance osteogenic differentiation of hDFCs. Hyperpolarizing cells by decreasing [K^+^]_e_ increased intracellular Ca^2+^ levels of hDFCs. Optogenetic activation of NpHR channels by an optogenetic system induced membrane hyperpolarization and significantly enhanced the proliferation, migration, and osteogenic differentiation abilities of NpHR-hDFCs. Conclusions: Hyperpolarization by optogenetic activation of NpHR channels can promote hDFCs’ proliferation, migration, and osteogenic differentiation abilities.

## 1. Introduction

Dental follicle cells (DFCs) are mesenchymal stem cells (MSCs) residing in dental follicle tissue and serve as precursor cells of periodontal tissues, possessing the capacity to form alveolar bone, periodontal ligament, and cementum. A large body of recent studies has demonstrated that DFCs hold promising application prospects and clinical translational value in tissue engineering [1]. Researchers have also been continuously exploring various approaches to enhance the biological performance of DFCs, such as combining different growth factors with biomaterial scaffolds to achieve improved bone repair efficiency [2]. However, because the molecular mechanisms underlying osteogenic differentiation of DFCs have not yet been fully elucidated and effective regulatory strategies for the biological characteristics of DFCs remain lacking, the application of DFCs in tissue engineering and their clinical translation still face considerable challenges.

Membrane potential are closely associated with stem cell differentiation. Osteogenic differentiation of MSCs is regulated by membrane potential [3,4], and the level of differentiation is positively correlated with membrane hyperpolarization [5]. Fischer-Lougheed, Liu, and colleagues reported that membrane hyperpolarization is a prerequisite for human myoblasts to initiate the differentiation process [6,7]. In addition, Komarova, Weidema, and colleagues reported that membrane hyperpolarization exerts a positive regulatory effect on the physiological functions of osteoclasts [8,9]. Ca^2+^ is crucial for stem cell differentiation, Ca^2+^ entry across the plasma membrane is a major source for MSCs to mobilize their Ca^2+^ content [10,11], and membrane potential is a key regulator of intracellular Ca^2+^ levels. Ca^2+^ influx is driven by the Ca^2+^ concentration gradient across the plasma membrane, which is modulated by the membrane potential. Membrane hyperpolarization increases the driving force for Ca^2+^ influx, whereas membrane depolarization decreases the driving force for Ca^2+^ influx [12,13]. For example, membrane depolarization converts physiological Ca^2+^ oscillations into sustained Ca^2+^ rises in T lymphocytes, as the driving force for Ca^2+^ entry through store-operated Ca^2+^ channels (SOCs) is reduced [14]. Kir2.1-mediated membrane hyperpolarization has been shown to raise cytosolic Ca^2+^, which is indispensable for myoblast differentiation to proceed [15]. Therefore, membrane potential changes can modulate intracellular Ca^2+^ levels by altering the electrochemical driving force for Ca^2+^ entry through ion channels in MSCs, given that these channels are in an open or activatable state.

Optogenetic technology based on light-activated channels represents a groundbreaking technique in the field of neuroscience in recent years. Optogenetics combines the advantages of spatiotemporal precision and non-invasiveness, enabling precise regulation of the electrophysiological activity of target cells through optical control, thereby achieving directed modulation of cellular biological functions. The principle of optogenetic technology lies in utilizing molecular biology, virology, and other approaches to introduce exogenous light-activated channel protein genes into living cells, express photosensitive channel proteins on the cell membrane, and control the activation and closure of these proteins through illumination with specific wavelengths of light, thereby altering the cell membrane potential, such as through depolarization and hyperpolarization. light-activated channels are mainly classified into excitatory and inhibitory channel proteins. Excitatory channel proteins are represented by ChR2 (channelrhodopsin-2), which mediates cation influx upon blue light illumination, leading to cell membrane depolarization. Inhibitory channel proteins are represented by Natronomonas pharaonis halorhodopsin (NpHR), which mediates anion influx upon yellow light illumination, leading to cell membrane hyperpolarization. Currently, optogenetics has become a mature technology and has been widely applied in the biomedical field. For example, by controlling different types of nerve cells, fundamental research on neural circuits can be conducted [16,17,18]; by directing the differentiation of stem cells from various sources into nerve cells, research on neural injury repair can be achieved [19,20].

Given the working principle of optogenetic technology, this study took human dental follicle cells (hDFCs) as the research subject. By constructing engineered hDFCs expressing the NpHR photosensitive channel, the effects of optogenetic activation of NpHR channel on membrane potential, proliferation, migration, and osteogenic differentiation of NpHR-hDFCs were investigated.

## 2. Materials and Methods

### 2.1. Cell Culture and Lentiviral Transduction

hDFCs were cultured from the dental follicle tissues obtained from third molar tooth germs with incomplete root development that required extraction for orthodontic treatment [21]. The Ethics Committee of the Affiliated Stomatology Hospital of the Southwest Medical University approved all human cell culture experiments (220815003).Tissue blocks were washed, minced into 1 mm^3^ fragments, and incubated in a solution of 1% collagenase and 1% dispase for 40 min at 37 °C. Both single cells and digested tissues were incubated in Dulbecco’s modified Eagle’s medium (DMEM; Gibco, Grand Island, NY, USA) supplemented with 10% fetal bovine serum (FBS), 100 units/mL penicillin, and 100 mg/mL streptomycin at 37 °C and 5% CO_2_. The culture medium was changed every 2 days before passage, and cells from passages 2 to 3 were used for experiments. After culturing for 5–7 days, elongated spindle-shaped cells adhering to the culture surface in a radial pattern were observed under microscopy. Cells were passaged upon reaching 80% confluence, and passages 2–3 (P2–P3) were used for the experiments.

The lentiviral vector carrying the NpHR photosensitive channel gene sequence (pLenti-CMV-eNpHR 3.0-EYFP-WPRE, titer: 1 × 10^11^ TU/L) was constructed and packaged (Cyagen, Guangzhou, China). The lentivirus was diluted 1:1000 in the culture medium, aliquoted, and stored at −80 °C. When cells reached 80% confluence, the transduction group was infected with the diluted lentivirus. At 48 h post-transduction, images were captured under a fluorescence microscope to confirm transduction efficiency.

### 2.2. Optogenetic Stimulation

Cells were subjected to light irradiation (580 nm, 1 mW/cm^2^) 48 h after transduction [22]. An LED lamp was used to irradiate the cells from above for 1 h per day. Control cells were maintained in a continuous dark environment.

### 2.3. Osteogenic Differentiation Induction, Alizarin Red Staining, and Alkaline Phosphatase (ALP) Staining

hDFCs were cultured in an osteogenic medium (DMEM media supplemented with 10% FBS, 10 mM sodium β-glycerophosphate, 10 nM dexamethasone, 50 μg·mL^−1^ ascorbic acid, and 10 nM vitamin D_3_). The induction medium was replaced every 3 days during the culture period. After continuous induction for 14 days, cells were fixed with 4% paraformaldehyde and subjected to alizarin red staining to observe mineralized nodule formation. For quantitative analysis, extraction was performed with 1 mL of 10% cetylpyridinium chloride solution, followed by shaking for 30 min. The microplate reader was set at a wavelength of 562 nm, with the detection mode set to single-wavelength absorbance.

For measurement of ALP activity, the ALP chromogenic working solution was freshly prepared according to the kit instructions (Beyotime Biotechnology, Shanghai, China). Staining was performed in the dark at room temperature using the chromogenic solution. After staining, cells were rinsed with PBS three times, and images were captured under a microscope.

To examine the effect of membrane depolarization on osteogenic differentiation, cells were subjected to osteogenic induction using a customized medium (Boster Biological Technology, Wuhan, China). All components were identical to those in the basal medium except that the K^+^ concentration was elevated to 50 mM, while the Na^+^ concentration was concurrently reduced to 95 mM to maintain osmotic balance and prevent osmotic stress.

### 2.4. Real-Time Reverse Transcriptase Polymerase Chain Reaction (RT-qPCR)

Total RNA was extracted from hDFCs using TRIzol reagent (Thermo Fisher Scientific, Waltham, MA, USA). After reverse transcription of total RNA into cDNA using a one-step RT-PCR kit (TaKaRa, Tokyo, Japan), RT-qPCR amplification was performed in a 20 μL reaction system. The amplification was carried out on an Applied Biosystems Prism 7900HT Sequence Detection System (Thermo Fisher Scientific, Waltham, MA, USA) with a PrimeScript RT-PCR Kit (TaKaRa, Tokyo, Japan). The program was set as follows, with fluorescence signal changes monitored throughout: pre-denaturation at 95 °C for 30 s; and PCR amplification at 95 °C for 5 s and 60 °C for 30 s for a total of 40 cycles. Fluorescence signals were collected after each cycle during this stage for quantitative analysis. Melting curve analysis was conducted at 95 °C for 15 s, 60 °C for 1 min, followed by a temperature increase to 95 °C at a rate of 0.5 °C/s, with continuous fluorescence signal acquisition. This was used to verify the specificity of amplification products and ensure the absence of primer dimers and non-specific amplification bands. All melting curves presented a single peak without any miscellaneous peaks. GAPDH was used as the internal reference, and the relative expression level of the target gene was calculated using the standard 2^−ΔΔCT^ methods. The primer sequences for the target gene NpHR were: F 5′-TCAACATCGCACTTGCAGGA-3′ and R 5′-CGCCAATCCAGTGTAGGAGG-3′; for RUNX2, F 5′-GCAGCAGCAGCAGCAGGAG-3′ and R 5′-GCACCGAGCACAGGAAGTTGG-3′; and for OCN, F 5′-GCCAGGCAGGTGCGAAGC-3′ and R 5′-GTCAGCCAACTCGTCACAGTCC-3′.

### 2.5. Cell Viability Assay (Cell Counting Kit-8, CCK-8)

Cells were seeded at a density of 3000 cells per well. After culturing for 1, 3, and 5 days, the CCK-8 kit (Beyotime, Shanghai, China) was used, and absorbance was measured at 450 nm using a microplate reader.

### 2.6. Transwell Migration Assay

Cells were serum-starved in an FBS-free medium for 12–24 h the day before plating. After digestion and counting, cells were diluted to 1–10 × 10^4^ cells/mL in a serum-free medium. A 24-well cell culture plate was used, and 500–600 μL of complete medium containing 10% FBS was added to the lower chamber of each well, ensuring a flat liquid surface without bubbles. Transwell chambers with a pore size of 8 μm were selected and rinsed once with the serum-free medium on both the upper and lower surfaces of the membrane to remove any impurities. Then, 200 μL of cell suspension was added to the upper chamber. The transwell chamber was slowly inserted into the 24-well plate and incubated in a 37 °C incubator for 24 h. The chamber was then removed, and the medium in the upper chamber was aspirated. After rinsing with PBS three times, cells were fixed and stained with 0.2% crystal violet, rinsed again, and observed and photographed under a microscope.

### 2.7. Wound Healing Assay

Three horizontal lines were drawn on the bottom of a six-well plate as reference marks. Cells were seeded at a density of 5 × 10^5^ cells per well according to the experimental groups. Two vertical lines perpendicular to the plate were scratched, and the detached cells were washed away with PBS. After replacing with the serum-free medium, images were immediately captured at 0 h as a control. After 24 h of culture, images were captured again. The scratch area was analyzed and quantified using ImageJ software (version 1.53k; National Institutes of Health, Bethesda, MD, USA).

### 2.8. Measurement of Intracellular Ca^2+^

Intracellular Ca^2+^ levels were measured with the ratiometric fluorescent dye Fura-2. Briefly,. the hDFCs attached to a sterile glass coverslip were loaded with Fura-2/AM (5 μM) for 30 min at room temperature. Subsequently, the coverslip with attached cells was transferred to a 1 mL chamber on the stage of a Leica inverted microscope (Leica, Wetzlar, Germany). The dye-loaded cells were gently perfused for 20 min with an external solution containing (mM) 130 NaCl, 1.8 CaCl_2_, 5.4 KCl, 1.0 MgCl_2_, 10 glucose, and 10 HEPES (pH 7.4). The TILLvisION 4.0 imaging system (TILL Photonic, Munich, Germany) connected to a cooled charge-coupled device camera was used for digital imaging of changes in intracellular Ca^2+^ in individual cells. Data were analyzed for intracellular Ca^2+^ changes (F340/F380) by measuring the excitation signals at 340 and 380 nm, and emission signal at 510 nm. The ratio of 340/380 was calculated by the ΔF/F0 method. To observe the effects of low [K^+^]_e_ on the intracellular Ca^2+^ levels of the cells, the basal external solution was changed to a solution containing low [K^+^]_e_.

### 2.9. Patch-Clamp Experiment

Whole-cell recordings were performed using an EPC-10 USB amplifier and PatchMaster software (version 2 × 91, HEKA Elektronik, Lambrecht, Germany). Patch-clamp microelectrodes with a resistance of 2.0–3.0 MΩ were used. The series resistance was less than 10 MΩ and compensated by at least 80% to minimize voltage errors. Whole-cell patch clamp was employed in current-clamp mode to record the cell membrane potential. The holding potential was set at −10 mV, near the physiological resting state of MSCs, which maintains the membrane in its native, unstimulated electrical condition. The extracellular solution composition (in mM) was: 135 NaCl, 2 CaCl_2_, 5.4 KCl, 1 MgCl_2_, 15 glucose, and 10 HEPES (pH = 7.4). The intracellular solution composition (in mM) was: 140 KCl, 1 MgCl_2_, 10 EGTA, 1 K_2_ATP, and 5 HEPES (pH = 7.4). The recorded electrophysiological data were analyzed using PatchMaster software.

### 2.10. Statistical Analysis

Experimental results are expressed as mean ± standard deviation. The t test was used to determine the differences between the two groups, and two-way ANOVA was used to determine the differences among multiple groups (GraphPad Software version 9.0, San Diego, CA, USA).

## 3. Results

### 3.1. Membrane Hyperpolarization Controls Osteogenic Differentiation of hDFCs by Increasing Intracellular Ca^2+^ Levels

**Figure 1:**
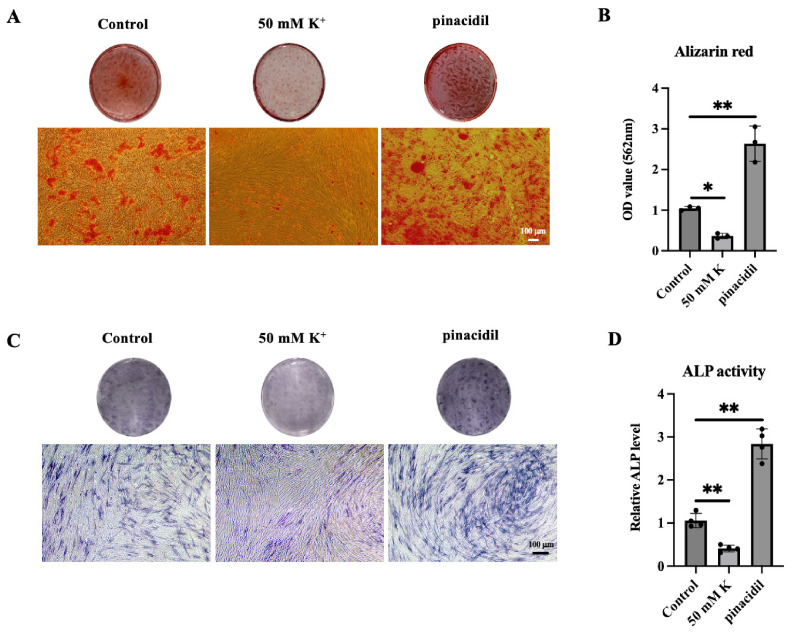
Membrane hyperpolarization controls osteogenic differentiation of hDFCs. The cells were induced in osteogenic medium for 14 days in the absence or presence of 50 mM [K^+^]_e_ or 10 mM pinacidil. (**A**) Mineralization of hDFCs was determined by Alizarin red staining. (**B**) Semi-quantitative analysis of Alizarin red staining at 562 nm. (**C**) ALP activity of hDFCs was determined by ALP staining. (**D**) Quantitative data for ALP staining. (**A**,**C**) Upper panel: observed by the naked eye; lower panel: observed by microscope (magnification: ×200). Osteogenic media served as a control group. Bars represent the mean ± SD, * *p* < 0.05, ** *p* < 0.01.

**Figure 2:**
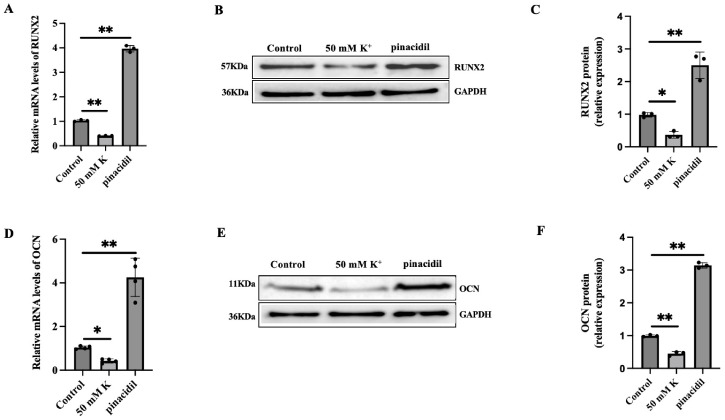
Membrane hyperpolarization controls osteogenic marker expression of hDFCs. The cells were induced in osteogenic medium for 14 days in the absence or presence of 50 mM [K^+^]_e_ or 10 mM pinacidil. (**A**) Gene expression levels of RUNX2 were determined utilizing RT-qPCR. (**B**) Protein expression of RUNX2 were determined using WB analysis. (**C**) Relative band density of (**B**). (**D**) Gene expression levels of OCN were determined utilizing RT-qPCR. (**E**) Protein expression of OCN were determined using WB analysis. (**F**) Relative band density of (**E**). Osteogenic media served as a control group. Bars represent the mean ± SD, * *p* < 0.05, ** *p* < 0.01.

We determined whether membrane hyperpolarization was functionally required for hDFCs osteogenic differentiation. For this purpose, hDFCs were osteogenically differentiated in the presence of high concentrations of extracellular K^+^ (50 mM [K^+^]_e_), which inhibits the membrane hyperpolarization of the cells, or in the presence of pinacidil, an ATP-sensitive K^+^ channel opener, activation of which promotes membrane hyperpolarization [4]. The Nernst equation confirms that equimolar substitution of NaCl with KCl (final concentrations: 95 mM NaCl and 50 mM KCl) induced about 53 mV membrane depolarization. As shown in Figure 1, after 14 days of osteogenic differentiation, a moderate formation of mineral deposits was observed in the control cells. Compared to this positive control, only a few mineral deposits were visible in the cells treated with 50 mM [K^+^]_e_, whereas a strong formation of mineral deposits was visible in the cells treated with 10 mM pinacidil (Figure 1A). This observation was verified by quantification of Alizarin red crystals (Figure 1B). ALP, OCN and RUNX2, as markers of osteogenic differentiation, are critical to osteogenesis and matrix mineralization. The results showed that ALP activity (Figure 1C,D) and the gene and protein expression of RUNX2 and OCN decreased in cells treated with 50 mM [K^+^]_e_, but increased with pinacidil treatment, compared to the positive controls (Figure 2A–F). Taken together, these data demonstrated that membrane hyperpolarization is essential for osteogenic differentiation of hDFCs.

**Figure 3:**
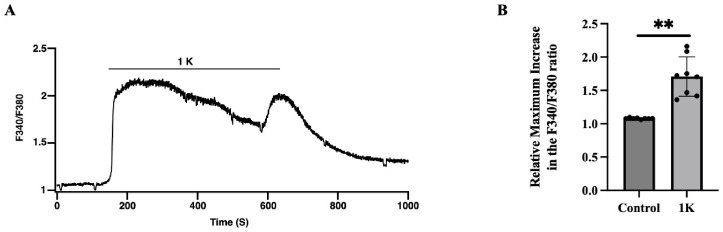
Low [K^+^]_e_-induced elevation of intracellular Ca^2+^ levels in hDFCs. (**A**) A representative F340/F380 trace reflecting the intracellular Ca^2+^ levels from hDFCs in response to 1 mM [K^+^]_e_. (**B**) Relative maximum increase in the F340/F380 ratio from hDFCs in response to 1 mM [K^+^]_e_. Bars represent the mean ± SD, ** *p* < 0.01.

Ca^2+^ is crucial for stem cell differentiation, and membrane potential is a key regulator of intracellular Ca^2+^ levels. Therefore, we next performed single-cell Ca^2+^ imaging to determine whether membrane hyperpolarization manipulates intracellular Ca^2+^ levels in hDFCs. Decreasing [K^+^]_e_ is a standard method of hyperpolarizing cells [23]. Therefore, we decreased [K^+^]_e_ to 1 mM and observed the effects of low [K^+^]_e_ on the intracellular Ca^2+^ levels of hDFCs. Based on the Nernst equation, 1 mM [K^+^]_e_ induced membrane hyperpolarization by approximately 44.0 mV. Perfusion with 1 mM [K^+^]_e_ significantly increased the intracellular Ca^2+^ levels of hDFCs (Figure 3A,B). These results demonstrated that membrane hyperpolarization promotes hDFCs’ osteogenic differentiation by increasing intracellular Ca^2+^ levels.

### 3.2. Effects of Optogenetic Activation of NpHR Channels on the Proliferation of hDFCs

**Figure 4:**
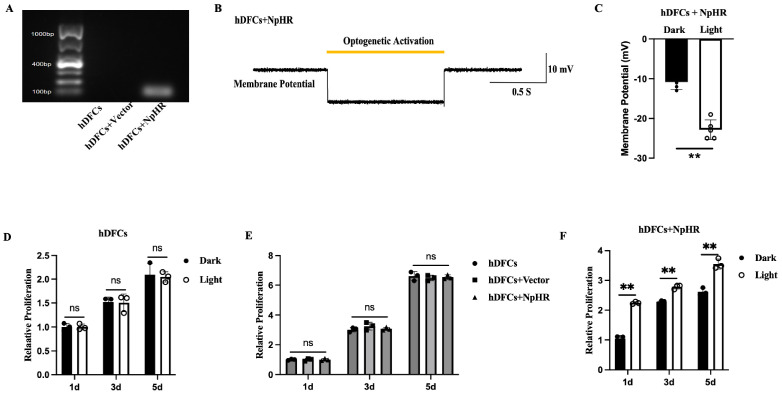
Light irradiation promoted proliferation of engineered hDFCs expressing NpHR channels. (**A**) Gene expression of NpHR channels in engineered hDFCs was verified by PCR. hDFCs and hDFCs transfected with empty lentiviral vectors (hDFCs + vector) worked as control. (**B**) Light irradiation induced membrane hyperpolarization in engineered hDFCs (hDFCs + NpHR). (**C**) Statistical data for (**B**). (**D**) The effects of light irradiation on the proliferation of hDFCs were determined using CCK8 assays. (**E**) The effects of transduction of NpHR lentiviral vectors or empty lentiviral vectors on the proliferation of hDFCs were determined using CCK8 assays. (**F**) The effects of light irradiation on the proliferation of engineered hDFCs (hDFCs + NpHR) were determined using CCK8 assays. Bars represent the mean ± SD, ** *p* < 0.01; ns, not significant.

Engineered hDFCs expressing NpHR channels (hDFCs + NpHR) were constructed through lentiviral transduction. The gene expression of NpHR in engineered hDFCs were verified by PCR (Figure 4A). The patch-clamp results showed that optogenetic activation of NpHR channels induced membrane hyperpolarization in NpHR-hDFCs (Figure 4B,C). We first evaluated the effects of optogenetic activation of NpHR channels on the proliferation of hDFCs. CCK-8 results showed that light irradiation, transduction of NpHR lentiviral vectors or empty lentiviral vectors had no effects on hDFCs’ proliferation (Figure 4D,F). Compared with the cells under dark conditions, light irradiation promoted the proliferative capacity of engineered hDFCs (Figure 4F). These results demonstrate that optogenetic activation of NpHR channels exert a promoting effect on the proliferation of hDFCs.

### 3.3. Effects of Optogenetic Activation of NpHR Channels on the Migration of hDFCs

**Figure 5:**
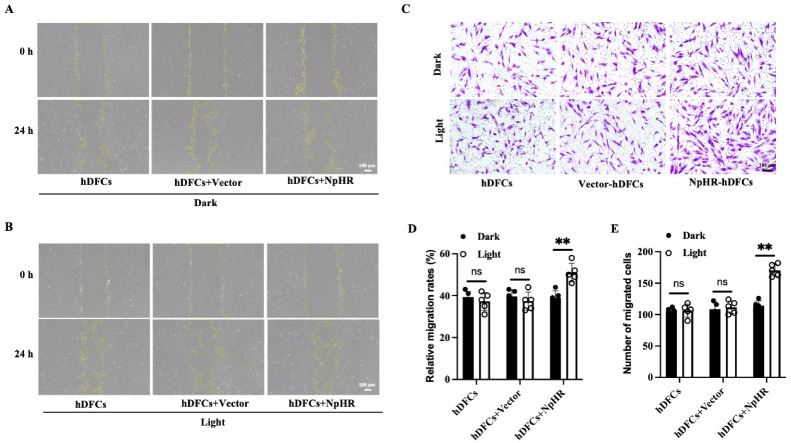
Light irradiation promoted migration of engineered hDFCs expressing NpHR channels. (**A**) The impact of transduction of NpHR lentiviral vectors or empty lentiviral vectors on hDFCs migration was assessed via a wound healing assay (×100 magnification). (**B**) The effect of light irradiation on the migration of hDFCs, hDFCs + vector, or hDFCs + NpHR was evaluated using a wound healing assay (×100 magnification). (**C**) Both the transduction effects of NpHR lentiviral vectors or empty lentiviral vectors and the impact of light irradiation on migration were examined using a transwell assay (×200 magnification). (**D**) Summarized data of (**A**,**B**). (**E**) Summarized data of (**C**). Data are presented as mean ± SD, ** *p* < 0.01; ns, not significant.

The effects of optogenetic activation of NpHR channels on the migration of hDFCs were evaluated by wound healing and transwell assays. Wound healing assay results showed that under dark conditions, there were no significant differences among the groups (Figure 5A). Compared with the cells under dark conditions, light irradiation promoted the migratory capacity of engineered hDFCs (Figure 5B,D). Consistent with the wound healing assay results, transwell assay results showed that under dark conditions, there were no statistically significant differences in the number of cells migrating to the lower chamber after 24 h of culture among the groups (Figure 5C). Compared with the cells under dark conditions, the number of engineered hDFCs migrating to the lower chamber was significantly increased upon light irradiation (Figure 5C,E). These results demonstrate that optogenetic activation of NpHR channels exert a promoting effect on the migration of hDFCs.

### 3.4. Effects of Optogenetic Activation of NpHR Channels on the Osteogenic Differentiation of hDFCs

**Figure 6:**
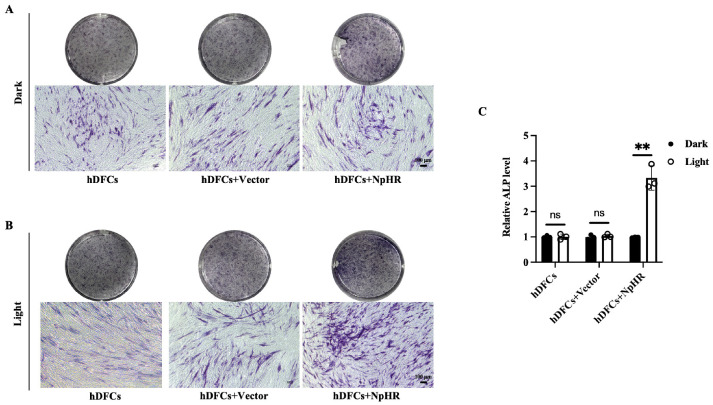
Light irradiation increased ALP activity of engineered hDFCs expressing NpHR channels. The cells were induced in osteogenic medium for 14 days. (**A**) The impact of transduction of NpHR lentiviral vectors or empty lentiviral vectors on ALP activity of hDFCs was assessed via a ALP assay (×100 magnification). (**B**) The effect of light irradiation on the ALP activity of hDFCs, hDFCs + vector, or hDFCs + NpHR was evaluated via a ALP assay (×100 magnification). (**C**) Quantitative data for ALP staining. Data are presented as mean ± SD, ** *p* < 0.01; ns, not significant.

**Figure 7:**
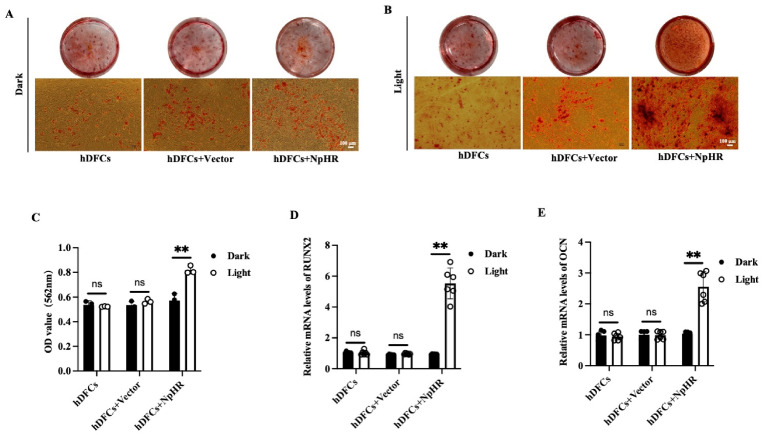
Light irradiation promoted osteogenic differentiation of engineered hDFCs expressing NpHR channels. The cells were induced in osteogenic medium for 14 days. (**A**) The impact of transduction of NpHR lentiviral vectors or empty lentiviral vectors on mineralization of hDFCs was determined by Alizarin red staining (×100 magnification). (**B**) The effect of light irradiation on mineralization of hDFCs, hDFCs + vector, or hDFCs + NpHR was evaluated via Alizarin red staining (×100 magnification). (**C**) Semi-quantitative analysis of Alizarin red staining at 562 nm. (**D**,**E**) Gene expression levels of RUNX2 and OCN were determined utilizing RT-qPCR. Bars represent the mean ± SD, ** *p* < 0.01; ns, not significant.

We evaluated the effects of optogenetic activation of NpHR channels on the osteogenic differentiation of hDFCs. After 14 days of osteogenic induction, ALP staining and alizarin red staining results showed that under dark conditions, there were no significant differences in ALP activity (Figure 6A,C) and mineralized nodule formation among the groups (Figure 7A,C). Compared with the cells under dark conditions, ALP activity was significantly increased (Figure 6B,C) and the number of mineralized nodules was significantly increased in engineered hDFCs upon light irradiation (Figure 7B,C). Consistent with the above results, RT-qPCR results showed that compared with the cells under dark conditions, the relative expression levels of osteogenesis-related genes (Runx2 and OCN) were significantly increased in engineered hDFCs upon light irradiation (Figure 7D,E). These results demonstrate that optogenetic activation of NpHR channels exert a promoting effect on the osteogenic differentiation of hDFCs.

## 4. Discussion

Membrane hyperpolarization is closely related to the differentiation capacity of stem cells. Membrane hyperpolarization is well correlated with higher levels of differentiation. Fischer-Lougheed, Liu, and colleagues reported that the occurrence of membrane hyperpolarization is a prerequisite for human myoblasts to initiate the differentiation process [6,7]. In addition, Komarova, Weidema, and colleagues reported that membrane hyperpolarization exerts a positive regulatory effect on the physiological functions of osteoclasts [8,9]. In the present study, we found that membrane hyperpolarization due to optogenetic activation of NpHR channels promoted the osteogenic differentiation of hDFCs, which is consistent with the above-mentioned findings.

Membrane potential is determined by transmembrane ion movement mediated by ion channels on the cell membrane. Cation influx mediated by ion channels leads to membrane depolarization, as seen with Ca^2+^ and Na^+^ channels, whereas cation efflux or anion influx mediated by ion channels, such as K^+^ and Cl^−^, leads to membrane hyperpolarization. To date, numerous studies have shown that MSCs express abundant K^+^ and Cl^−^ channels [24]. Further studies have indicated that K^+^ or Cl^−^ channels are involved in the regulation of proliferation and migration of MSCs. For instance, membrane hyperpolarization mediated by Cl^−^ influx through Cl^−^ channels promote the proliferation of MSCs [25]. Membrane hyperpolarization mediated by K^+^ channels is involved in the regulation of cell migration. For example, Hu et al. found that KV2.1 regulates the directional migration and homing of MSCs [26]. NpHR is a light-driven Cl^−^ inward pump that is widely used as an optogenetic tool. In the present study, we found that optogenetic activation of NpHR channels induced membrane hyperpolarization in engineered hDFCs and promoted both proliferation and migration. These findings are consistent with previous research results.

In the tissue regeneration applications and stem cell therapy of MSCs, the balance between proliferation and differentiation is an important issue to be addressed. On the one hand, MSCs maintain cellular homeostasis through proliferation in vivo; on the other hand, MSCs can acquire bone tissue function through direct differentiation into osteoblasts and osteocytes [27]. During the osteogenic differentiation of MSCs, it is essential not only to ensure a sufficient number of cells but also to prevent cellular senescence caused by excessive expansion, which would impair the proliferative and differentiation capacities of MSCs [28]. Therefore, in this study, we utilized an optogenetic system to promote the proliferation and migration of hDFCs without compromising their osteogenic differentiation capacity.

An increase in intracellular Ca^2+^ level is a frequently observed phenomenon during stem cell differentiation [29]. To investigate the mechanisms by which membrane hyperpolarization regulates hDFCs osteogenic differentiation, we performed single-cell Ca^2+^ imaging to observe the effects of low [K^+^]_e_ on the intracellular Ca^2+^ level of hDFCs. We found that membrane hyperpolarization caused by low [K^+^]_e_ induced an increase in the intracellular Ca^2+^ level of hDFCs. These results demonstrate that membrane hyperpolarization elevated intracellular Ca^2+^ levels in hDFCs. Supporting this, it has been reported that membrane hyperpolarization resulting from Kir2.1 channels increases intracellular Ca^2+^ and that this step is essential to allow myoblast differentiation to proceed [15]. Although we have demonstrated in this study that membrane hyperpolarization can elevate intracellular Ca^2+^ levels, we wish to emphasize that hyperpolarization merely increases the electrochemical driving force for Ca^2+^ influx, and this effect is only manifested when the relevant channels are in an open or activatable state. The driving force for Ca^2+^ entry is a necessary electrochemical prerequisite, but it does not itself trigger Ca^2+^ influx. As Behringer et al. reported, hyperpolarization alone did not alter intracellular Ca^2+^ under control conditions; however, it significantly increased Ca^2+^ in the presence of acetylcholine, when the relevant channels were activated [12]. This finding underscores that Ca^2+^ influx requires both a favorable electrochemical driving force and the opening/activation of Ca^2+^-permeable channels.

## 5. Limitations

This study utilized an optogenetic system to promote the proliferation and migration of hDFCs while maintaining their osteogenic differentiation capacity, providing novel insights for periodontal tissue regeneration. However, this study has the following limitations. First, we acknowledge that the mechanism by which hyperpolarization increases intracellular Ca^2+^ in hDFCs requires further investigation. While the driving force itself does not trigger channel opening, resting cells may exhibit basal activity of Ca^2+^-permeable channels. We also do not exclude the possibility that hDFCs express hyperpolarization-activated Ca^2+^ channels. In addition, it has been reported that low [K^+^]_e_ may also elevate intracellular Ca^2+^ via alternative pathways, such as regulating Na^+^/K^+^-ATPase and Na^+^/Ca^2+^ exchangers [30]. Therefore, the conclusion in this study that membrane hyperpolarization leads to elevated intracellular Ca^2+^ levels remains to be further validated by other membrane potential intervention approaches. Second, the study has not yet been fully integrated with clinical oral practice. The local inflammatory microenvironment, hypoxic conditions, and bacterial products in periodontal bone defects may affect the response efficiency of the optogenetic system; the precise delivery methods of light stimulation within the confined oral space, the type of light source (such as LED or optical fiber), and the optimization of irradiation parameters (wavelength, intensity, duration) remain to be explored. Second, this study was primarily based on in vitro cell experiments without in vivo animal validation. The in vivo transduction efficiency of optogenetic elements, the effects of light stimulation on surrounding tissues, and the actual repair efficacy in complex periodontal defect models remain unclear. Future research should focus on addressing these limitations to facilitate the clinical translation of this technology.

## 6. Conclusions

Hyperpolarization by optogenetic activation of NpHR channels can promote hDFCs proliferation, migration, and osteogenic differentiation abilities.

## Data Availability

The datasets used and/or analyzed during the current study are available from the corresponding author on reasonable request.
